# The effect of body size evolution and ecology on encephalization in cave bears and extant relatives

**DOI:** 10.1186/s12862-017-0976-1

**Published:** 2017-06-05

**Authors:** Kristof Veitschegger

**Affiliations:** Palaeontological Institute and Museum, University of Zurich, Karl Schmid-Strasse 4, 8006 Zürich, Switzerland

**Keywords:** Physiological buffer, Dormancy, Diet, *Ailuropoda*, *Helarctos*, *Melursus*, *Tremarctos*, *Ursus*

## Abstract

**Background:**

The evolution of larger brain volumes relative to body size in Mammalia is the subject of an extensive amount of research. Early on palaeontologists were interested in the brain of cave bears, *Ursus spelaeus*, and described its morphology and size. However, until now, it was not possible to compare the absolute or relative brain size in a phylogenetic context due to the lack of an established phylogeny, comparative material, and phylogenetic comparative methods. In recent years, many tools for comparing traits within phylogenies were developed and the phylogenetic position of cave bears was resolved based on nuclear as well as mtDNA.

**Results:**

Cave bears exhibit significantly lower encephalization compared to their contemporary relatives and intraspecific brain mass variation remained rather small. Encephalization was correlated with the combined dormancy-diet score. Body size evolution was a main driver in the degree of encephalization in cave bears as it increased in a much higher pace than brain size. In *Ursus spelaeus*, brain and body size increase over time albeit differently paced. This rate pattern is different in the highest encephalized bear species within the dataset, *Ursus malayanus*. The brain size in this species increased while body size heavily decreased compared to its ancestral stage.

**Conclusions:**

Early on in the evolution of cave bears encephalization decreased making it one of the least encephalized bear species compared to extant and extinct members of Ursidae. The results give reason to suspect that as herbivorous animals, cave bears might have exhibited a physiological buffer strategy to survive the strong seasonality of their environment. Thus, brain size was probably affected by the negative trade-off with adipose tissue as well as diet. The decrease of relative brain size in the herbivorous *Ursus spelaeus* is the result of a considerable increase in body size possibly in combination with environmental conditions forcing them to rest during winters.

**Electronic supplementary material:**

The online version of this article (doi:10.1186/s12862-017-0976-1) contains supplementary material, which is available to authorized users.

## Background

Cave bears, *Ursus spelaeus*, were a common faunal element during the Pleistocene of Europe and Asia [1]. The habitat of *U. spelaeus* was Eurasia with an east-west extension ranging from Spain to the Altai Region of Russia [1–3]. The ancestral species of *U. spelaeus*, *U. deningeri*, was even more widespread, with a habitat ranging from Spain to Siberia and even reaching the British Isles [1, 3–5]. At the end of the Pleistocene, cave bears shared the same fate as most other elements of the Pleistocene megafauna and became extinct [6–8]. Their time of extinction was proposed to be around 27.800–25.000 years BP [9, 10]. Based on molecular data, the sister group to cave bears are brown bears, *U. arctos*, and polar bears, *U. maritimus*, together (Fig. 2). The evolutionary lineage of *U. spelaeus* split from these two bear species sometime between 2.75 to 1.2 Ma years ago [11–13]. Traditionally, cave bears were considered to be predominantly or exclusively herbivorous based on the morphology of their teeth and jaws [1, 14–18]. Several studies presented isotopic as well as morphometric evidence confirming this hypothesis [2, 19–25]. However, the predominantly herbivorous diet of cave bears was questioned based on isotopic [26, 27], morphometric [28, 29], microwear [30, 31], and taphonomic evidence [32]. In recent years, many of these studies were dismissed based on methodological errors or repeated with the result that cave bears were indeed herbivorous [2, 19, 20, 33].

Cave bear brains are among the earliest ones of an extinct species to be investigated and several studies discuss different aspects of its evolution [34–42]. Many of these studies focus on the external morphology of artificial, fossil, or virtual endocasts [34, 35, 39–41]. Conflicting statements were presented concerning the overall size of the cave bear brain. Some authors suggested a small brain size compared to body size and speculated that the increase of skull size in the evolution of *U. spelaeus* outpaced brain size [35, 36]. Others suggested high brain volumes for cave bears and an opposite scenario with brain size outpacing body size [37, 38, 42]. Many factors affect the size of brains. Brain tissue itself is known to be expensive to produce and maintain [43–45]. Absolute as well as relative brain size can be influenced by social structure [46–48], environment [48–52], sensory systems [53], evolutionary history [54–57], body size evolution [42], and different physiological as well as life history trade-offs [43, 52, 57–66].

Diet can have a profound effect on brain size as was exemplified in bats and primates [67]. Recently, it was even suggested that diet had a bigger effect on brain size than sociality in primates [68]. The diet of bears is diverse with varying amounts of plant and animal matter within and among species [2]. It ranges from hypercarnivorous in polar bears, *U. maritimus*, to folivorous in giant pandas, *Ailuropoda melanoleuca* [2, 69]. Thus, diet of bears might exhibit a link to brain size.

Some bear species survive the cold seasons with extended resting periods, whereas especially tropical species are active year-round [69]. Resting periods in bears are different from deep hibernation as movement still can occur [70]. Thus, these periods are better described as dormancy in bears. Previous to dormancy, bears increase the amount of stored body fat [70]. The storage of high amounts of adipose tissue was linked to a decreased brain size [60]. Bears represent a good study object to investigate the effect of dormancy on brain size because some species are active year round whereas others increase the amount of adipose tissue annually [69].

In this study, I investigate the absolute and relative brain size of *U. spelaeus* and all extant bear species in a phylogenetic context and add remarks on *U. deningeri*. For this, I created a comprehensive brain size dataset for all extant bear species and cave bears. Additionally, I examine potential variables which could introduce energetic constraints affecting brain size evolution such as dormancy, diet, and body size. These variables were chosen because they can be reconstructed for cave bears with some measure of certainty.

## Methods

### Data collection

Altogether, I measured 412 skulls of 10 extant and extinct bear species (Table 1). *U. spelaeus* samples cover a time period of about 20.000 years based on radiocarbon dating [9]. Brain volume was measured using the glass bead method [71]. I used 6 mm diameter soda lime glass beads. The individual body mass (g) was inferred using the basicranial length (SKL) as described by van Valkenburgh: body mass (kg) = 2.02*Log10(SKL)-2.80 (least squares regression) [72]. Brain volume was converted into brain mass (g) using the specific weight of brain substance 1.036 (g/cm3) [73]. The collected data is presented in Additional file 1: Table S1. To assess the validity of previously published cranial volumes of cave bears, I additionally created a data subset predicting endocranial volume based on external skull measurements for *U. spelaeus*, *U. arctos*, and *U. malayanus* [74]. Raw data for this analysis can be found in Additional file 2: Table S2.

**Table 1:** Results of body mass (g) and brain mass (g) estimates as well as residuals and investigated ecological scores

Species	n	average body mass (g)	StD body mass (g)	average body mass literature (g)	average brain mass (g)	StD brainmass (g)	average residuals	StD average residuals	diet score	dormancy score	d*d
*Ailuropoda melanoleuca*	5	118'637 (105'324–135'094)	10,748.36	97'500 (70'000–125'000)	281.79 (238.28–331.52)	33.89	−0.0029	0.0548	1.000	3.000	3.000
*Tremarctos ornatus*	8	80'918 (64'223–110'621)	15,049.56	117'500 (60'000–175,000)	227.92 (176.12–279.72)	31.33	0.0373	0.0320	1.814	3.000	5.443
*Ursus americanus*	28	117'116 (83'885–155'600)	20,168.42	170'000 (40'000–300'000)	256.78 (186.48–352.24)	38.39	−0.0373	0.0422	1.884	1.000	1.884
*Ursus arctos*	93	177'628 (92'655–320'042)	40,696.57	390'000 (55'000–725'000)	378.08 (207.20–538.72)	61.38	−0.0080	0.0464	1.637	1.000	1.637
*Ursus deningeri*	1	254,996	-	-	341.88	-	−0.1770	-	-	-	-
*Ursus malayanus*	50	82'379 (56'333–108'841)	13,709.85	52'500 (25'000–80'000)	340.43 (227.92–435.12)	47.31	0.2047	0.0403	2.684	3.000	8.051
*Ursus maritimus*	82	211'265 (144'141–277'270)	33,275.87	402'500 (150'000–655'000)	498.80 (393.68–611.24)	53.75	0.0525	0.0320	2.970	2.000	5.940
*Ursus spelaeus*	99	322'764 (209'553–425'411)	57,207.28	362'500 (225'000–500'000)	430.10 (321.16–569.80)	52.36	−0.1550	0.0443	1.000	1.000	1.000
*Ursus thibetanus*	29	113'424 (78'533–166'402)	21,401.65	120'000 (40'000–200'000)	282.58 (186.48–414.40)	45.66	0.0155	0.0577	1.920	1.000	1.920
*Ursus ursinus*	17	147'081 (124'439–183'291)	18,122.18	100'000 (50'000–150'000)	292.52 (248.64–352.24)	26.04	−0.0573	0.0360	2.606	3.000	7.818

The materials examined in this study are from the following collections: Biologiezentrum Linz (BZL), Geology School of Aristotle University Thessaloniki (AUTH), Institut für Paläontologie Wien (PIUW), Naturalis Biodiversity Center Leiden (NBC), Naturhistorisches Museum der Burgergemeinde Bern (NMBE). Naturhistorisches Museum Wien (NHM), Naturmuseum St. Gallen (NMSG), Naturmuseum Südtirol Bozen (PZO), Muséum National d’Histoire Naturelle Paris (MNHN), Museum für Naturkunde Berlin (MfN), Paleontological Institute and Museum University of Zurich (PIMUZ), and Zoological Museum University of Zurich (ZMUZH).

### Data analyses

**Fig. 1:**
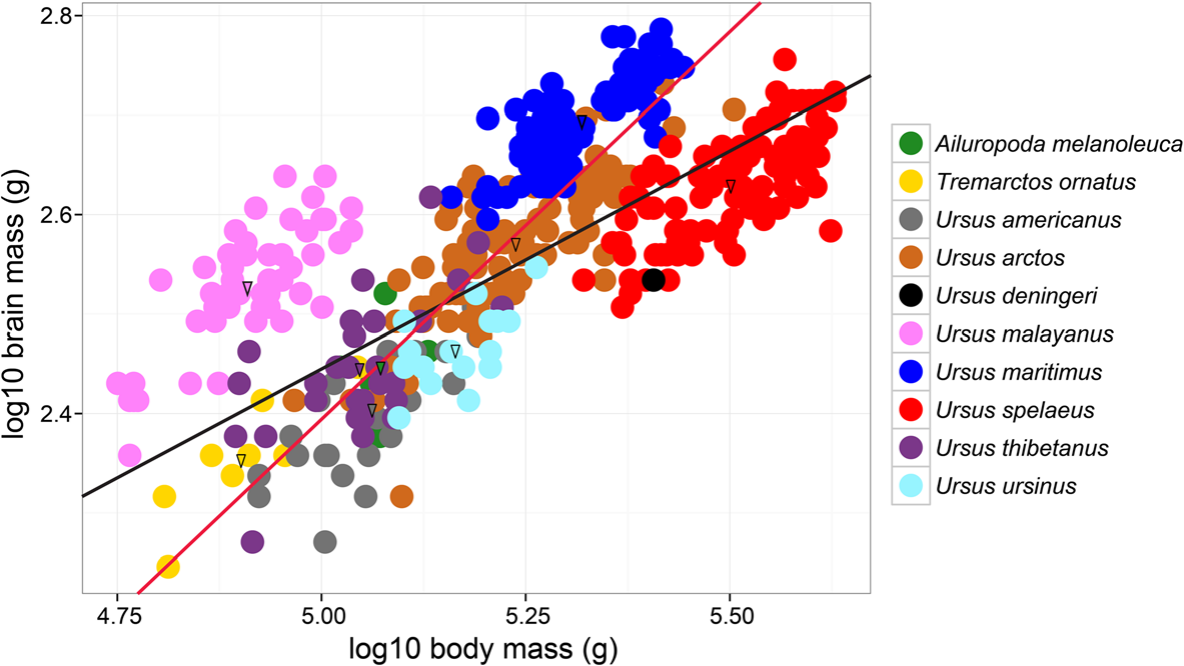
Scatterplot of log10 brain mass (g) against log10 body mass (g) with a PGLS regression lines (phylogenetic generalized least squares). In black is the PGLS regression line for all data points (*p* value: 0.0148, slope: 0.43978, intercept: 0.24623, adjusted R^2^: 0.5378), in red the PGLS regression line without *Ursus malayanus* and *Ursus spelaeus* (*p* value: 0.0016, slope: 0.78069, intercept: −1.50995, adjusted R^2^: 0.8606). The triangles represent the mean for each species on which PGLS was calculated

Data were log10-transformed and examined using ordinary least squares (OLS) and phylogenetic generalized least squares (PGLS) (Fig. 1, Additional file 3: Supplementary Information). I used OLS to investigate the differences in intercepts and slopes between species. Residuals from a PGLS based on brain/body mass (g) were used to investigate the differences in relative brain size. With this, the data were corrected for the effect of size. An initial investigation revealed that the data were heavily skewed by *U. malayanus* and *U. spelaeus* because of the uneven sampling (Additional file 3: Supplementary Information). All other bear species were more similar in body mass (g)/brain mass (g). This was supported by the multiple and adjusted R^2^ (Additional file 3: Supplementary Information). Thus, the basis for brain/body mass (g) residuals was the slope (0.78069) and intercept (−1.50995) as retrieved by a PGLS excluding *U. malayanus* and *U. spelaeus*. For PGLS, the species-averaged brain mass and body mass were used. Analyses were performed in R, version 3.2.3 [75]. PGLS was executed as implemented in the packages ape and caper [76, 77]. Results from OLS regressions on all data points as well as a PGLS regression with all species are presented in the Additional file 3: Supplementary Information.

The phylogenetic relationships among Ursidae is not completely understood as there are clear discrepancies between trees based on nuclear and mitochondrial DNA (mtDNA), mirroring a complex evolutionary history with introgression and incomplete lineage sorting [78]. Complete phylogenies of Ursidae including cave bears are based on mtDNA [11, 12], therefore, I use mtDNA topology as basis for the phylogenetic analyses. The relationship between cave bears and brown bear as well as polar bear was also confirmed by nuclear DNA [79]. Recently, several new, former unrecognized species and subspecies of *U. spelaeus* were described based on morphological and genetic data [3, 80–83]. However, some of these taxa are polyphyletic [84, 85]. Here, I include all these proposed cave bear species and subspecies in *U. spelaeus,* but exclude the well-established ancestral species *U. deningeri* [17]. *U. deningeri* is considered an anagenetic ancestor to *U. spelaeus* [1, 80] and thus was excluded from all analyses as it would either represent a duplication or cannot be properly placed in phylogeny. Branch lengths for phylogenetically informed analyses were retrieved from Nyakatura and Bininda-Emonds [86] and Bon [12].

Due to uneven sampling and small sample sizes in species-averaged datasets, I use non-parametric analyses. A Kruskal-Wallis test followed by a Dunn’s test with Bonferroni adjustment was used on the resulting residuals to test for significant differences. This was performed in R, version 3.2.3 [75], using the packages pgirmess and PMCMR [87, 88]. The subset of different brain volume estimations was analysed using a Wilcoxon signed-rank test for paired samples in R, version 3.2.3. [75]. Boxplots were created in the package ggplot2 [89].

I used squared-change parsimony [90] to reconstruct ancestral stages for log 10 average body mass (g), log 10 average brain mass (g), and averaged residuals respectively. This analysis follows a Brownian motion model of evolution [91]. The resulting ancestral character states were then used to investigate the relative mass change (in percent) from one node to the following within the tree. These analyses were performed for each variable separately in Mesquite software (version 3.01) [92].

To test for a possible effect of dormancy and diet on relative and absolute brain size, I scored each of these variables between 1 and 3: 1 represents states where a smaller brain size is expected and 3 the opposite. Dormancy was scored as 1 (dormancy), 2 (fasting periods), and 3 (no dormancy) [69]. Dietary preferences were scored using the compilation from van Heteren et al. [2]. The diet was scored between 1 (completely folivorous/low caloric diet) to 3 (completely faunivorous (high caloric diet) using the formula:

$$ \mathrm{Diet}\ \mathrm{score}={\left(\mathrm{percent}\ \mathrm{folivory}/\mathrm{overall}\ \mathrm{percent}\right)}^{\ast }1+{\left(\mathrm{percent}\ \mathrm{frugivory}/\mathrm{overall}\ \mathrm{percent}\right)}^{\ast }2+{\left(\mathrm{percent}\ \mathrm{faunivory}/\mathrm{overall}\ \mathrm{percent}\right)}^{\ast }3 $$

The scoring enables to multiply both scores to one under the assumption that unidirectional or opposing trends show a combined effect on brain size. This is possible because the array of possible variables is constrained among three states. I performed the Kendall’s tau correlation analysis in R, version 3.2.3, using the package Kendall [93].

## Results

The resulting averaged reconstructed body mass (g) and brain mass (g) with standard deviation as well as the ecological scores are given in Table 1.

The slopes of the OLS regression lines of the different bear species were not significantly different from each other. Intercepts, however, were in many cases significantly different among species (Table 2, Additional file 4: Table S3). The intercept of cave bears was not significantly different from that of *U. americanus* and *U. ursinus*.

**Table 2:** Results of the pairwise comparisons of slopes and intercepts among different bear species

	*Ailuropoda melanoleuca*	*Tremarctos ornatus*	*Ursus americanus*	*Ursus arctos*	*Ursus malayanus*	*Ursus maritimus*	*Ursus spelaeus*	*Ursus thibetanus*	*Ursus ursinus*
*Ailuropoda melanoleuca*		+/− 0.0034	+/− 0.0366	+/− 0.0306	+/− 0.1725*****	+/− 0.1086*****	+/− 0.0596***	+/− 0.0129	+/− 0.0347
*Tremarctos ornatus*	+/− 0.4053		+/− 0.0400**	+/− 0.0273	+/− 0.1691*****	+/− 0.1052*****	+/− 0.0630***	+/− 0.0096	+/− 0.0380*
*Ursus americanus*	+/− 0.3957	+/− 0.0096		+/− 0.0672*****	+/− 0.2091*****	+/− 0.1452*****	+/− 0.0230	+/− 0.0495*****	+/− 0.0019
*Ursus arctos*	+/− 0.3362	+/− 0.0691	+/− 0.0595		+/− 0.1419*****	+/− 0.0779*****	+/− 0.0903*****	+/− 0.0177	+/− 0.0653*****
*Ursus malayanus*	+/− 0.3895	+/− 0.0158	+/− 0.0062	+/− 0.0533		+/− 0.0639*****	+/− 0.2321*****	+/− 0.1596*****	+/− 0.2072*****
*Ursus maritimus*	+/− 0.2864	+/− 0.1189	+/− 0.1093	+/− 0.0498	+/− 0.1031		+/− 0.1682*****	+/− 0.0956*****	+/− 0.1432*****
*Ursus spelaeus*	+/− 0.2161	+/− 0.1892	+/− 0.1796	+/− 0.1200	+/− 0.1734	+/− 0.0702		+/− 0.0726*****	+/− 0.0250
*Ursus thibetanus*	+/− 0.2497	+/− 0.1556	+/− 0.1460	+/− 0.0865	+/− 0.1398	+/− 0.0367	+/− 0.0335		+/− 0.0476*****
*Ursus ursinus*	+/− 0.1606	+/− 0.2447	+/− 0.2351	+/− 0.1756	+/− 0.2289	+/− 0.1258	+/− 0.0555	+/− 0.0891	

Significant results are marked with stars (*p*-value: *< 0.5, **< 0.1, ***< 0.01, ****< 0.001, *****< 0.0001)

Upper triangle shows intercept comparisons and lower triangle shows slope comparisons

**Fig. 2:**
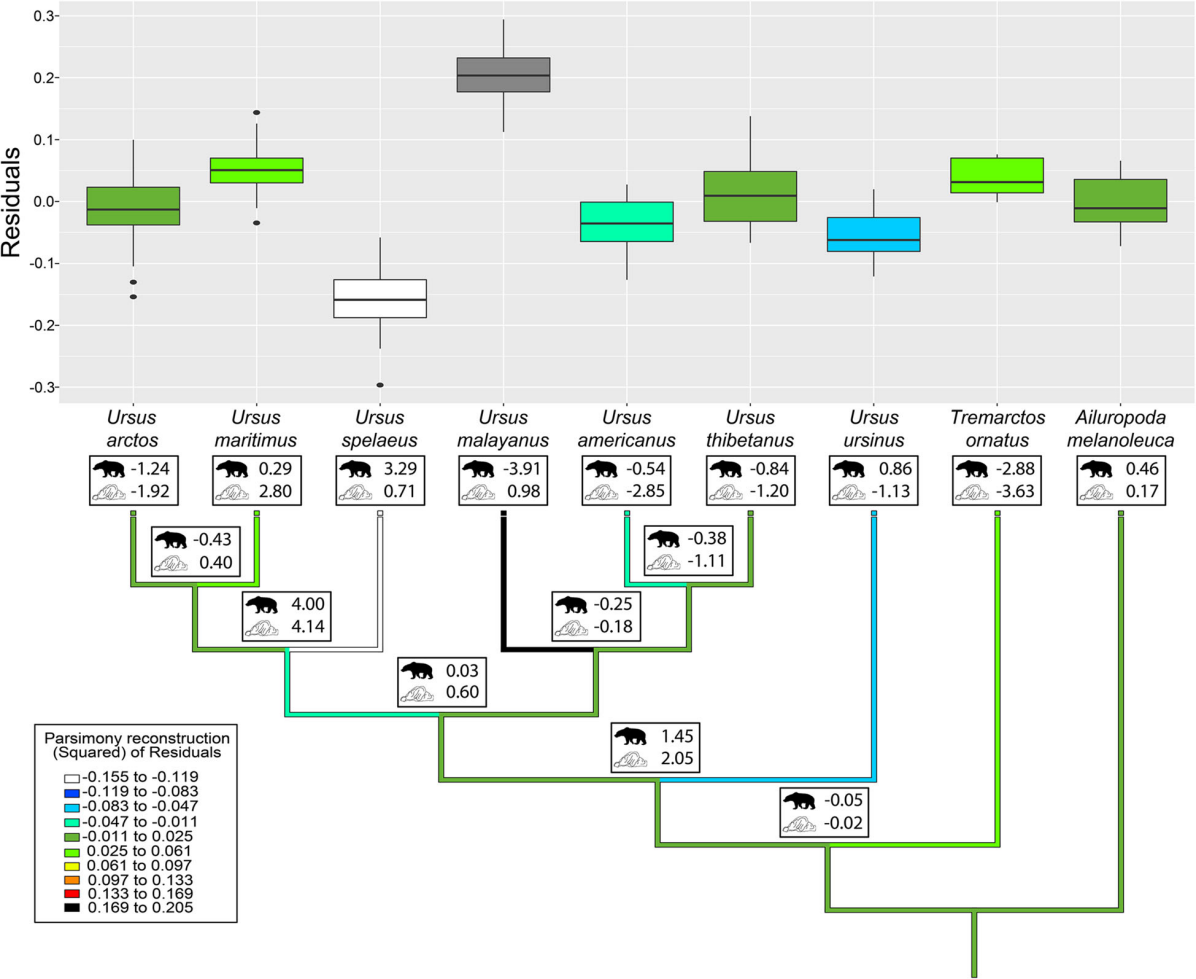
Boxplots of the distribution of the residuals from PGLS (excluding *Ursus malayanus* and *Ursus spelaeus*) for Ursidae as well as result of the squared change parsimony analysis. Additionally, the relative change (in percent) of log10 body mass (g) and log10 brain mass (g) is shown in the boxes for every node. Terminal root value for log10 body size is 5.05 (112,052 g) and for log10 brain size 2.44 (277 g)

*U. spelaeus* and *U. deningeri* have the lowest average residuals within the dataset, followed by *U. ursinus* and *U. americanus* (Fig. 2, Table 1). The highest average residuals were found in *U. malayanus* and *U. maritimus*. The Kruskal-Wallis test followed by a Dunn’s test with Bonferroni adjustment revealed that the residuals of *U. spelaeus* are significantly smaller than of most other bear species, except for *U. ursinus* and *A. melanoleuca* (Table 3).

**Table 3:** Results of the Kruskal-Wallis rank sum test on the residuals of investigated bear species

Kruskal-Wallis rank sum test								
K-W chi-squared: 338.89 df: 8, *p*-value: <0.0001	*Ailuropoda melanoleuca*	*Tremarctos ornatus*	*Ursus americanus*	*Ursus arctos*	*Ursus malayanus*	*Ursus maritimus*	*Ursus thibetanus*	*Ursus ursinus*
*Tremarctos ornatus*	1.0000	-	-	-	-	-	-	-
*Ursus americanus*	1.0000	0.7560	-	-	-	-	-	-
*Ursus arctos*	1.0000	1.0000	1.0000	-	-	-	-	-
*Ursus malayanus*	0.0699	0.4557	**<0.0001**	**<0.0001**	-	-	-	-
*Ursus maritimus*	1.0000	1.0000	**<0.0001**	**<0.0001**	**0.0006**	-	-	-
*Ursus thibetanus*	1.0000	1.0000	0.8645	1.0000	**<0.0001**	0.7134	-	-
*Ursus ursinus*	1.0000	0.2586	1.0000	1.0000	**<0.0001**	**<0.0001**	0.2484	-
*Ursus spelaeus*	0.1133	**<0.0001**	**0.0005**	**<0.0001**	**<0.0001**	**<0.0001**	**<0.0001**	0.2602

In bold are significant results

The biggest documented cave bear brain volume is 1.8 times bigger than the smallest. In comparison, in *U. arctos* it is 2.6 times bigger and in *U. thibetanus* 2.2 times. Polar bears, however, exhibit low variation with the biggest brain volume being 1.6 times bigger than the smallest (Table 1).

**Fig. 3:**
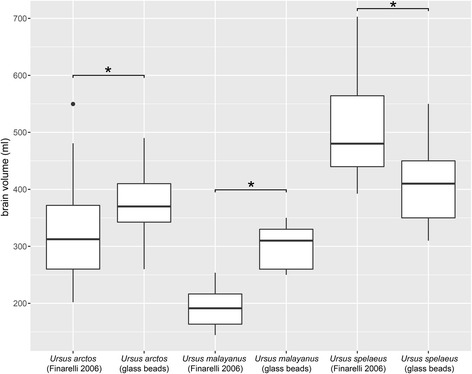
Comparison between two methods for estimating brain volumes of Ursidae (asterisks mark significant differences based on a Wilcoxon signed-rank test)

The comparison between different methods to estimate brain volumes revealed that external measurements produced results significantly different from brain volume measured directly with glass beads (Fig. 3). In *U. spelaeus*, brain volumes inferred by external measurements were significantly higher than those measured with soda lime glass beads (*n* = 15, median glass beads = 410 ml, median external measurements = 480 ml, V = 120, *p*-value = <0.0001). The opposite is true for *U. arctos* and *U. malayanus*. Here, brain volumes were significantly higher when measured with glass beads (*U. arctos*: *n* = 34, median glass beads = 370 ml, median external measurements = 312 ml, V = 66, *p*-value = <0.0001; *U. malayanus*: *n* = 9, median glass beads = 310 ml, median external measurements = 191 ml, V = 0, *p*-value = 0.0039).

The ancestral stage reconstruction based on squared-change parsimony revealed that the small relative brain size of *U. spelaeus* and *U. ursinus* represent a secondarily derived condition, as their respective ancestral stages exhibit a higher relative brain size (Fig. 2, Additional file 5: Table S4). The comparison between the relative change of body mass (g) and brain mass (g) shows that the evolution of a bigger body size in *U. spelaeus* outpaced brain size evolution. Both increased size compared to their ancestral stages, respectively; however, body size increased at a much higher pace. The reverse was found in *U. maritimus,* in which brain size evolution outpaced body size increase. Nonetheless, in *U. maritimus* and *U. spelaeus* brain as well as body size evolution are unidirectional towards increasing. In *U. americanus* the trend is unidirectional towards decrease. These cases contrast with the decoupling trend recorded for *U. malayanus*. In this species, the body size decreases where the brain size increases leading to the high relative brain size found in this species. At the basis of the tree, the analysis retrieved an ancestral body mass of 112,052 g and a brain mass of 277 g.

Using Kendall’s tau to find correlations between ecological scores and brain mass (g) revealed no significant results. Residuals were not significantly correlated with dormancy or diet scores. However, residuals were correlated with the combined score (Table 4).

**Table 4:** Results of Kendall’s tau on different scores as well as the combination of both

	**Diet score (d)**		**Dormancy score (d)**		d*d	
	tau	*p*-value	tau	*p*-value	tau	*p*-value
**Average brain mass (g)**	0.1970	0.5294	−0.1360	0.7285	0.0000	1.0000
**Residuals**	0.3660	0.2084	0.3400	0.2976	**0.5560**	**0.0476**

In bold are significant correlations

## Discussion

### Encephalization in Ursidae

*U. spelaeus* had a significantly smaller relative brain size than most extant bear species. The brain size variation in cave bears over time, between males and females [1] as well as high altitude and lowland populations [81] did not exceed the intraspecific variation in extant *U. americanus*, *U. arctos*, *U. malayanus*, and *U. thibetanus*. Especially, the relative brain size of *U. arctos* and *U. thibetanus* exhibits a considerable amount of variation. The study of brain size evolution often focuses on the evolution of increased encephalization and intelligence [38, 94–99]. Animals with bigger relative brain size often show more flexibility in behaviour and are potentially more adaptable [100–104]. Nonetheless, brain tissue is expensive and producing it comes at the cost of a slower life history [43–45, 57, 64, 105]. Therefore, in some species a secondary reduction of relative or absolute brain size was described [106]. Especially, islands represent a challenging habitat for many mammals and several species exhibit a secondary decrease in encephalization [107, 108]. Dormancy and diet, separately, were not correlated with brain size; however, the combination of both variables showed a significant effect. A possible explanation for this correlation could be that cave bears underwent a change in diet in a habitat in which they were still forced to rest during winters [1, 9] limiting the possibility of so called cognitive buffering [66, 109]. Under the Cognitive Buffer hypothesis, it is expected that relative brain size of mammals in highly seasonal environment increases due to the necessity of behavioural flexibility. This, however, also implies an active reaction towards the environmental change. In contrast, dormancy does not require this high level of behavioural flexibility but relies on body fat storage, which additionally has a negative trade-off with brain size [60, 66]. This suggests that brain size in cave bears might exhibit a physiological buffering effect [66] partly constraining relative brain size. Other bear species such as *U. arctos* and *U. americanus* would also exhibit this physiological buffering effect but their food quality or life history might lessen the constraint on relative brain size.

In Ursidae, three life history variables have been demonstrated to correlate with encephalization: gestation time (negative), newborn mass (positive), and litter size (negative) [57]. In *A. melanoleuca,* a combination of these variable with a year-round active strategy [69] is potentially the reason why the second herbivorous species in the dataset exhibits an encephalization higher than found in cave bears. Nonetheless, the life history correlates with encephalization are not unidirectional in the giant panda. In contrast, the highest encephalized species, *U. malayanus*, shows unidirectional trends towards increased encephalization in most variables with heavy newborns, small litter size, non-resting strategy, and 68% faunivory [2, 69, 110]. Gestation time and litter size are not known for *U. spelaeus*. However, cave bears were about the same size as *U. arctos* at birth [14, 111], contributing to its small relative brain size. A small relative brain size can already be traced in *U. deningeri*. This ancestor of *U. spelaeus* also exhibits low encephalization and is usually considered a herbivorous species with winter resting behaviour as well [25, 112].

The effect of diet alone on brain size in Ursidae remains elusive. In other groups such as primates and bats the link is more apparent. Fruit, blood, and meat eating bats tend to be more encephalized than their insect-eating relatives and in primates leaf-eaters are the least encephalized [67, 68]. Although a comparable link was proposed for Carnivora, it is hypothesized to be more associated with the process of acquiring food rather than the energetics of the diet itself [54, 67]. The change in diet in cave bears and associated smaller relative brain size is reminiscent of the often mentioned evolutionary arms-race between Carnivora and Ungulates in which Carnivora had to be more encephalized to outsmart their (herbivorous) prey [98]. This scenario, however, was later found to be unsubstantiated [113].

Smaers et al. [42] suggested that absolute brain size in the evolution of *U. spelaeus* was outpacing body size. This pattern was based on brain size estimates obtained by external measurements [37, 38, 74]. Although external measurements can predict brain volume with a certain confidence [74, 114], they can also have considerable prediction error [114]. The results of this study show that external measurements overestimate the endocranial volume of *U. spelaeus* (Fig. 3). The reason for this might be the frontal bossing found in cave bears likely caused by an extension of the frontal sinuses [16, 17, 35, 36, 41]. My results show that in cave bears body size evolution outpaced brain size evolution. Thus attesting to a remark by Marinelli [36]. Smaers et al. [42] also published brain and body size variables for three other extinct bear species *Arctodus simus* (3 Ma – 0.01 Ma)*, Cephalogale ursinus* (23.8 Ma – 22.8 Ma)*,* and *Indarctos oregonensis* (10.3 Ma – 5.3 Ma). With these values *C. ursinus* would be placed high above the regression line (residual: 0.33), *A. simus* close to the line (residual: 0.04), and *I. oregonensis* below the line (residual: −0.16). Fossil evidence has shown to change the results of suggested bidirectional evolution in brain size [99, 106]. However, in ursids, the cave bear lineage represents one of the least encephalized compared to extant and most extinct relatives.

### On the methodology of body mass reconstruction

I calculated the mass of every specimen individually based on skull length [72]. My body mass estimates, generally, were well within the range of known body mass distribution for each species (Table 1) [69]. However, the estimations for polar bears, *U. maritimus*, are generally small. Thus, this animal might be closer to the range of other bear species such as *U. arctos* in the scatterplot (Fig. 1). It is, nonetheless, possible that the measured skulls are from individuals from the lower range of mass distribution of this species. The opposite is true for the two smaller bear species *U. malayanus* and *U. ursinus*. *U. malayanus* potentially could have even bigger brains compared to body size than in the presented dataset. *U. ursinus* would be within the range of other extant bear species in the scatterplot (Fig. 1) such as *U. arctos*. *U. spelaeus* is considered to be one of the biggest carnivorans [115], with some estimates suggesting it to have surpassed the size of the polar bear or the Kodiak brown bear, *U. a. middendorffi*, by reaching a body mass of about 1′500 kg [1]. Based on this, the cave bear could have had an exceptionally small relative brain size. Considering the possible bias body mass estimations based on skull length had on the dataset, encephalization in Ursidae could be more even with two strong outliers, *U. malayanus* towards increased encephalization and *U. spelaeus* towards decreased one.

## Conclusion

The aim of this study was to examine the encephalization in cave bears and comparing it with living and extinct members of Ursidae. *U. spelaeus*, and subsequently all potential species associated with this taxon, exhibit one of the lowest encephalization in Ursidae because body size increase outpaced brain size increase in its evolution. This is a trend observable early on in the cave bear lineage as is evidenced by the low encephalization of *U. deningeri.* My results stand in contrast to previous interpretations of cave bear brain evolution [42]. I showed that this study has used overestimated brain volumes due to the shape of cave bear skulls. Bear species, which do not exhibit dormancy and have a high caloric diet, showed a weak but significant correlation with bigger relative brain size. This would be in accordance with the trait-off between brain size and adipose tissue as well as studies on diet and brain size [60, 66–68]. The ecological shift towards a plant based diet alone did not affect encephalization in cave bears. However, a more general link associated with food acquirement strategy might still exist [67]. The herbivorous *U. spelaeus* has a small relative brain size possibly due to the combined effect of unequal body/brain size evolution and a seasonal environment in which dormancy was necessary for survival.

## Additional files

Additional file 1:Table S1. Basicranial length, body mass estimates, brain volumes and brain mass for all examined bear skulls. (XLSX 33 kb)

Additional file 2: Table S2. Data subset of brain volume estimates based on external measurements by Finarelli [74] and glass bead method. (XLSX 12 kb)

Additional file 3: Supplementary Information. Results for different linear models and corresponding graphical output as well as boxplot on residuals based on PGLS with all species. (PDF 658 kb)

Additional file 4: Table S3. Additional results for slope and intercept pairwise comparisons. (XLSX 31 kb)

Additional file 5: Table S4. Node values for ancestral stage reconstructions. (XLSX 13 kb)
