# Supplementary material for: The effect of body size evolution and ecology on encephalization in cave bears and extant relatives
Source: BMC Evol Biol. 2017 Jun 5;17:124. doi: 10.1186/s12862-017-0976-1 (PMC5460516; doi:10.1186/s12862-017-0976-1)
Supplement: Supplementary file 3 — Results for different linear models and corresponding graphical output as well as boxplot on residuals based on PGLS with all species. (PDF 658 kb) [file 12862_2017_976_MOESM3_ESM.pdf]

Supplementary Information: The effect of body size evolution and ecology on encephalization in cave bears and extant relatives (Kristof Veitschegger)

Supplementary Table 4: Results of the different linear models investigated in this study.

**Model**	**Intercept**	**Std. Error**	**t**	**p**	**Slope**	**Std. Error**	**t**	**p**	**multiple R2**	**adjusted R2**
OLS (individual datapoints)	0.6268	0.0946	6.6250	**<0.0001**	0.3716	0.0180	20.6070	**<0.0001**	0.5088	0.5076
OLS (individual datapoints), without *Ursus malayanus* and *Ursus spelaeus*	-1.6605	0.1274	-13.0400	**<0.0001**	0.8113	0.0245	33.1700	**<0.0001**	0.8089	0.8081
PGLS	0.2462	0.7039	0.3498	0.7368	0.4398	0.1370	3.2108	**0.0148**	0.5956	0.5378
PGLS, without *Ursus malayanus* and *Ursus spelaeus*	-1.5100	0.6477	-2.3314	0.0671	0.7807	0.1266	6.1678	**0.0016**	0.8838	0.8606

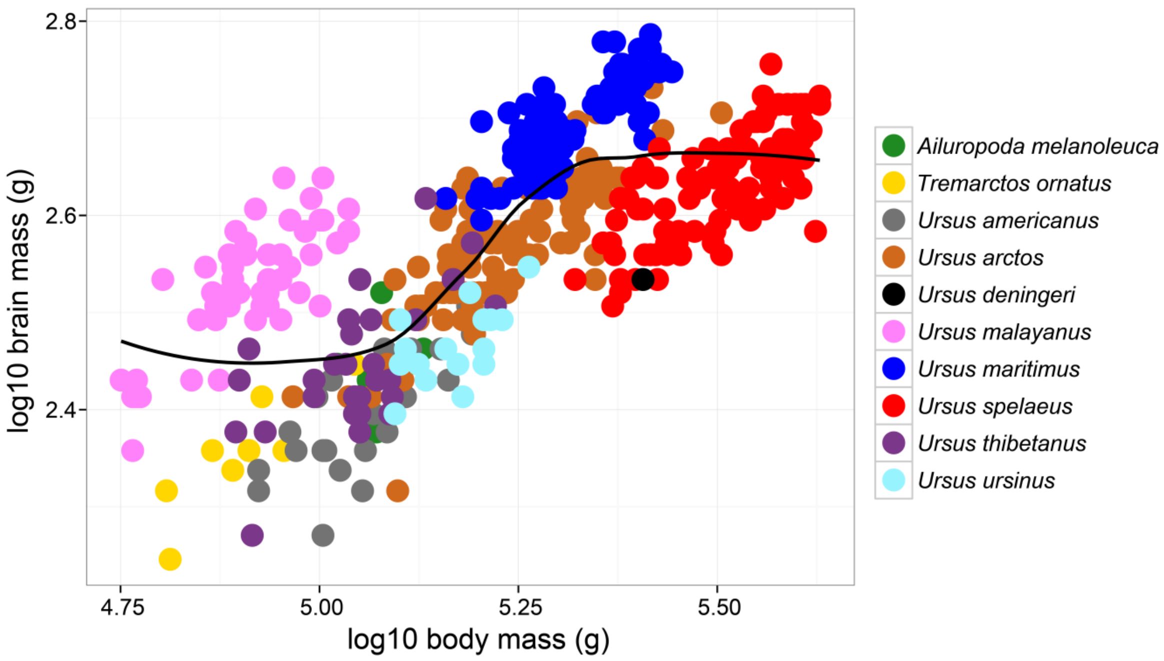

Supplementary Figure 1: Scatterplot of log10 brain mass (g) against log10 body mass (g) with a LOESS curve (local polynomial regression). Note that *Ursus malayanus* and *Ursus spelaeus* heavily skew the line in opposite directions, thus introducing bias to the linear model. Other bear species fall on one line.

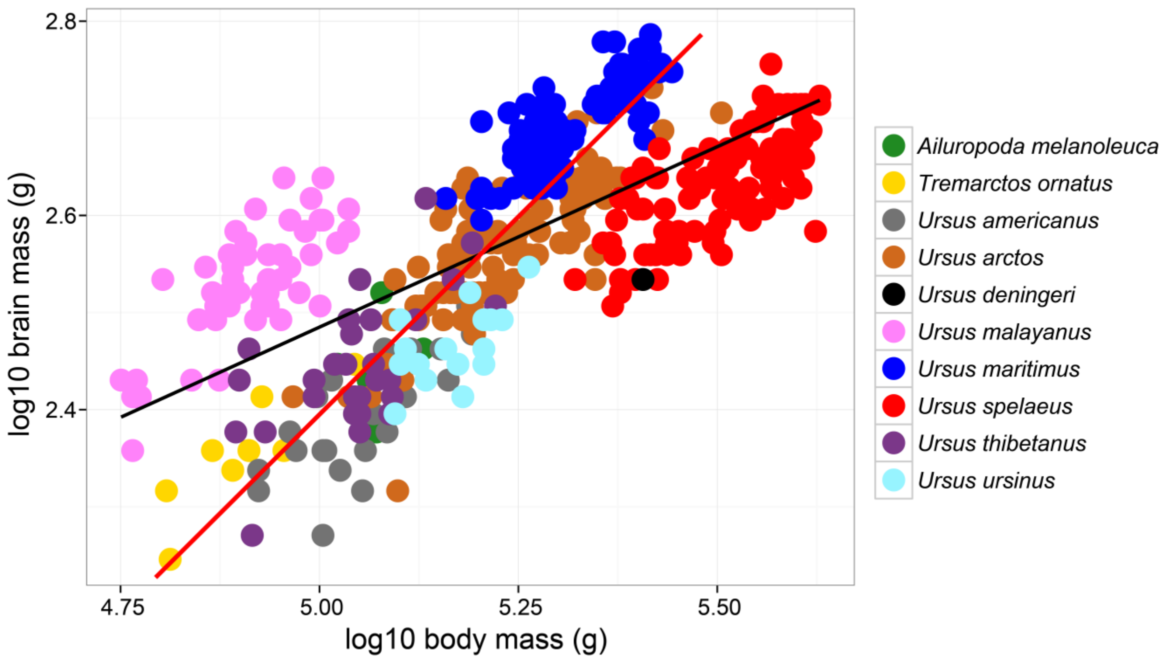

Supplementary Figure 2: Scatterplot of log10 brain mass (g) against log10 body mass (g) with a OLS regression lines (ordinary least squares). In black is the OLS regression line for all data points, in red the OLS regression line without *Ursus malayanus* and *Ursus spelaeus.* (details in Supplementary Table 4)

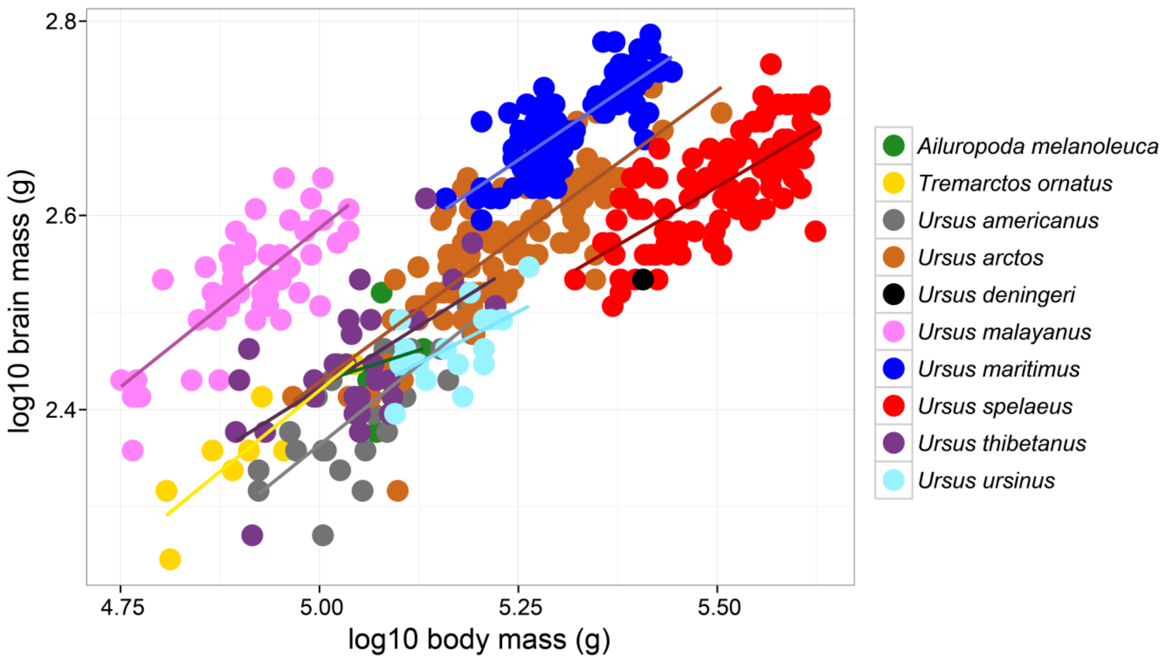

Supplementary Figure 3: Scatterplot of log10 brain mass (g) against log10 body mass (g) with OLS regression lines (ordinary least squares) for each species.

Supplementary Table 5: Slopes and intercepts based on OLS for all bear species as presented in Supplementary Figure 3.

**Model**	**Intercept**	**Std. Error**	**t**	**p**	**Slope**	**Std. Error**	**t**	**p**	**multiple R2**	**adjusted R2**
Ailuropoda melanoleuca	1.1051	3.8413	0.2880	0.7920	0.2646	0.7572	0.3490	0.7500	0.0391	-0.2812
Tremarctos ornatus	-0.9296	0.7951	-1.1690	0.2867	0.6699	0.1622	4.1300	**0.0061**	0.7398	0.6965
Ursus americanus	-0.9378	0.5494	-1.7070	0.0997	0.6603	0.1085	6.0850	**<0.0001**	0.5875	0.5716
Ursus arctos	-0.5754	0.2382	-2.4160	**0.0177**	0.6008	0.0455	13.2160	**<0.0001**	0.6575	0.6537
Ursus malayanus	-0.6836	0.3712	-1.8420	0.0717	0.6541	0.0756	8.6520	**<0.0001**	0.6093	0.6012
Ursus maritimus	-0.2354	0.2416	-0.9740	0.3330	0.5510	0.0454	12.1300	**<0.0001**	0.6478	0.6434
Ursus spelaeus	-0.0146	0.2652	-0.0550	0.9560	0.4807	0.0482	9.9750	**<0.0001**	0.5064	0.5013
Ursus thibetanus	-0.1497	0.6304	-0.2370	0.8141	0.5143	0.1249	4.1180	**0.0003**	0.3858	0.3630
Ursus ursinus	0.2687	0.7664	0.3510	0.7308	0.4252	0.1484	2.8650	**0.0118**	0.3537	0.3106

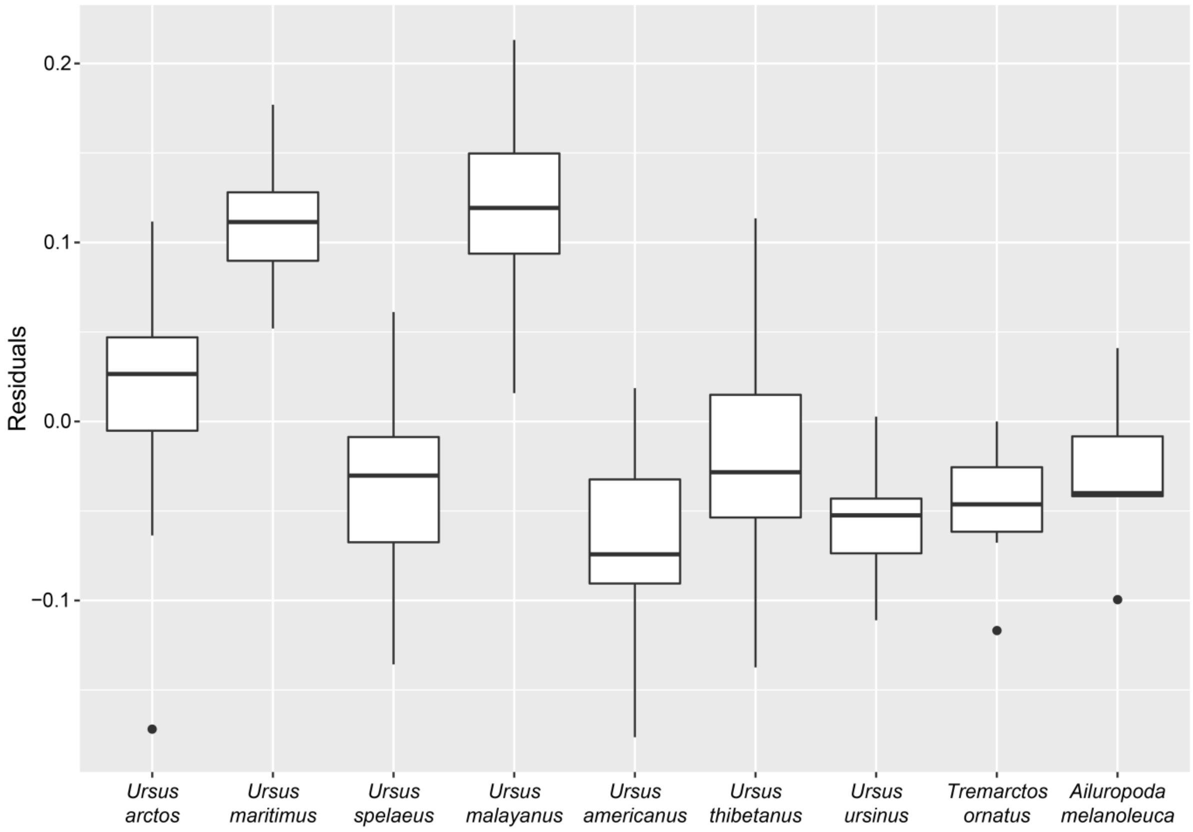

Supplementary Figure 4: Distribution of residuals based on PGLS (phylogenetic generalized least squares) on all species. Note the higher values for *Ursus spelaeus* compared to the main analysis.
